# Efficient sequential and parallel algorithms for planted motif search

**DOI:** 10.1186/1471-2105-15-34

**Published:** 2014-01-31

**Authors:** Marius Nicolae, Sanguthevar Rajasekaran

**Affiliations:** 1 Department of Computer Science and Engineering, University of Connecticut, Storrs, CT, USA

## Abstract

**Background:**

Motif searching is an important step in the detection of rare events occurring in a set of DNA or protein sequences. One formulation of the problem is known as (*l*,*d*)-motif search or Planted Motif Search (PMS). In PMS we are given two integers *l* and *d* and *n* biological sequences. We want to find all sequences of length *l* that appear in each of the input sequences with at most *d* mismatches. The PMS problem is NP-complete. PMS algorithms are typically evaluated on certain instances considered challenging. Despite ample research in the area, a considerable performance gap exists because many state of the art algorithms have large runtimes even for moderately challenging instances.

**Results:**

This paper presents a fast exact parallel PMS algorithm called PMS8. PMS8 is the first algorithm to solve the challenging (*l*,*d*) instances (25,10) and (26,11). PMS8 is also efficient on instances with larger *l* and *d* such as (50,21). We include a comparison of PMS8 with several state of the art algorithms on multiple problem instances. This paper also presents necessary and sufficient conditions for 3 *l*-mers to have a common *d*-neighbor. The program is freely available at http://engr.uconn.edu/~man09004/PMS8/.

**Conclusions:**

We present PMS8, an efficient exact algorithm for Planted Motif Search. PMS8 introduces novel ideas for generating common neighborhoods. We have also implemented a parallel version for this algorithm. PMS8 can solve instances not solved by any previous algorithms.

## Background

This paper presents an efficient exact parallel algorithm for the Planted Motif Search (PMS) problem also known as the (*l*,*d*) motif problem [1]. A string of length *l* is caller an *l*-mer. The number of positions where two *l*-mers *u* and *v* differ is called their Hamming distance and is denoted by *Hd*(*u*,*v*). For any string *T*, *T*[ *i*..*j*] is the substring of *T* starting at position *i* and ending at position *j*. The PMS problem is the following. Given *n* sequences *S*_1_,*S*_2_,…,*S*_*n*_ of length *m* each, from an alphabet *Σ* and two integers *l* and *d*, identify all *l*-mers *M*,*M*∈*Σ*^*l*^, that occur in at least one location in each of the *n* sequences with a Hamming distance of at most *d*. More formally, *M* is a motif if and only if ∀*i*,1≤*i*≤*n*,∃*j*_*i*_,1≤*j*_*i*_≤*m*-*l*+1, such that *Hd*(*M*,*S*_*i*_[*j*_*i*_..*j*_*i*_+*m*-1])≤*d*.

The PMS problem is essentially the same as the Closest Substring problem. These problems have applications in PCR primer design, genetic probe design, discovering potential drug targets, antisense drug design, finding unbiased consensus of a protein family, creating diagnostic probes and motif finding (see e.g., [2]). Therefore, efficient algorithms for solving the PMS problem are very important in biology and bioinformatics.

A PMS algorithm that finds all the motifs for a given input is called an exact algorithm. All known exact algorithms have an exponential worst case runtime because the PMS problem is NP-complete [2]. An exact algorithm can be built using two approaches. One is sample driven: for all (*m*-*l*+1)^*n*^ possible combinations of *l*-mers coming from different strings, generate the common neighborhood. The other is pattern-driven: for all *Σ*^*l*^ possible *l*-mers check which are motifs. Many algorithms employ a combination of these two techniques. For example, [3] and [4] generate the common neighbors for every pair of *l*-mers coming from two of the input strings. Every neighbor is then matched against the remaining *n*-2 input strings to confirm or reject it as a motif. Other algorithms ([5,6]) consider groups of three *l*-mers instead of two.

PMS algorithms are typically tested on instances generated as follows (also see [1,4]): 20 DNA strings of length 600 are generated according to the i.i.d. (independent identically distributed) model. A random *l*-mer is chosen as a motif and planted at a random location in each input string. Every planted instance is modified in at most *d* positions. For a given integer *l*, the instance (*l*,*d*) is defined to be challenging if *d* is the smallest integer for which the expected number of motifs of length *l* that occur in the input by random chance is ≥1. Some of the challenging instances are (13,4),(15,5),(17,6),(19,7),(21,8),(23,9),(25,10), (26,11), etc.

The largest challenging instance solved up to now has been (23,9). To the best of our knowledge the only algorithm to solve (23,9) has been qPMS7 [5]. The algorithm in [7] can solve instances with relatively large *l* (up to 48) provided that *d* is at most *l*/4. However, most of the well known challenging instances have *d*>*l*/4. PairMotif [3] can solve instances with larger *l*, such as (27,9) or (30,9), but these are significantly less challenging than (23,9). Furthermore, for instances that current algorithms have been able to solve, the runtimes are often quite large.

In this paper we propose a new exact algorithm, PMS8, which can efficiently solve both instances with large *l* and instances with large *d*. The efficiency of PMS8 comes mainly from reducing the search space by using the pruning conditions presented later in the paper, but also from a careful implementation which utilizes several speedup techniques and emphasizes cache locality.

One of the basic steps employed in many PMS algorithms (such as PMSprune [4], PMS5 [8], PMS6 [9], and qPMS7 [5]) is that of computing all the common neighbors of three *l*-mers. In qPMS7, this problem is solved using an Integer Linear Programming (ILP) formulation. In particular, a large number of ILP instances are solved as a part of a preprocessing step and a table is populated. This table is then repeatedly looked up to identify common neighbors of three *l*-mers. This preprocessing step takes a considerable amount of time and the lookup table calls for a large amount of memory. In this paper we offer a novel algorithm for computing all the common neighbors of three *l*-mers. This algorithm eliminates the preprocessing step. In particular, we don’t solve any ILP instance. We also don’t employ any lookup tables and hence we reduce the memory usage. We feel that this algorithm will find independent applications. Specifically, we state and prove necessary and sufficient conditions for 3 *l*-mers to have a common neighbor.

## Methods

For any *l*-mer *u* we define its *d*-neighborhood as the set of *l*-mers *v* for which *Hd*(*u*,*v*)≤*d*. For any set of *l*-mers *T* we define the common *d*-neighborhood of *T* as the intersection of the *d*-neighborhoods of all *l*-mers in *T*. To compute common neighborhoods, a natural approach is to traverse the tree of all possible *l*-mers and identify the common neighbors. A pseudocode is given in Appendix 1. A node at depth *k*, which represents a *k*-mer, is not explored deeper if certain pruning conditions are met. Thus, the better the pruning conditions are, the faster will be the algorithm. We discuss pruning conditions in a later section.

PMS8 consists of a sample driven part followed by a pattern driven part. In the sample driven part we generate tuples of *l*-mers originating from different strings. In the pattern driven part we generate the common *d*-neighborhood of such tuples. Initially we build a matrix *R* of size *n*×(*m*-*l*+1) where row *i* contains all the *l*-mers in *S*_*i*_. We pick an *l*-mer *x* from row 1 of *R* and push it on a stack. We filter out any *l*-mer in *R* at a distance greater than 2*d* from *x*. Then we pick an *l*-mer from the second row of *R* and push it on the stack. We filter out any *l*-mer in *R* that does not have a common neighbor with the *l*-mers on the stack; then we repeat the process. If any row becomes empty, we discard the top of the stack, revert to the previous instance of *R* and try a different *l*-mer. If the stack size is above a certain threshold (see section on Memory and Runtime) we generate the common *d*-neighborhood of the *l*-mers on the stack. For each neighbor *M* we check whether there is at least one *l*-mer *u* in each row of *R* such that *Hd*(*M*,*u*)≤*d*. If this is true then *M* is a motif. The pseudocode of PMS8 is given in Appendix 1.

A necessary and sufficient condition for 3 *l*-mers to have a common neighbor is discussed in the section on pruning conditions. For 4 or more *l*-mers we only have necessary conditions, so we may generate tuples that will not lead to solutions. However, due to the way the filtering works, the following observations apply. Let the stack at any one time contain *tl*-mers: *r*_1_,*r*_2_,…,*r*_*t*_ where *r*_*t*_ is at the top of the stack. Evidently, the *l*-mers on the stack pass the necessary pruning conditions for *tl*-mers. However, the following are also true. For any two *l*-mers *r*_*i*_ and *r*_*j*_ on the stack, there exists a common neighbor. This is true because whenever we filter an *l*-mer we make sure it has at least one neighbor in common with the *l*-mer at the stop of the stack. Thus, we can prove by induction that any two *l*-mers in the stack have a common neighbor. Second, any triplet of the form *r*_1_,*r*_2_,*r*_*i*_, 2<*i*≤*t*, has at least one common neighbor. This is true because when the stack had only the first two *l*-mers we filtered out any *l*-mer which doesn’t make a compatible triplet with the two. In general, for any number *p*<*t* the (*p*+1) tuples of the form *r*_1_,*r*_2_,…,*r*_*p*_,*r*_*i*_ where *p*<*i*≤*t* must pass the pruning conditions for *p*+1*l*-mers. Therefore, even though the pruning for more than 3 *l*-mers is not perfect, the algorithm implicitly tests the pruning conditions on many subsets of the *l*-mers in the stack and thus decreases the number of false positive tuples generated.

### Speedup techniques

#### Sort rows by size

An important speedup technique is to reorder the rows of *R* by size after every filtering step. This reduces the number of tuples that we consider at lower stack sizes. These tuples require the most expensive filtering because as the stack size increases, fewer *l*-mers remain to be filtered.

#### Compress *l*-mers

We can speed up Hamming distance operations by compressing all the l-mers of *R* in advance. For example, for DNA we store 8 characters in a 16 bit integer, divided into 8 groups of 2 bits. For every 16 bit integer *i* we store in a table the number of non-zero groups of bits in *i*. To compute the Hamming distance between two *l*-mers we first perform an exclusive or of their compressed representations. Equal characters produce bits of 0, different characters produce non-zero bits. Therefore, one table lookup provides the Hamming distance for 8 characters. One compressed *l*-mer requires *l*∗⌈log|*Σ*|⌉ bits of storage. However, we only need the first 16 bits of this representation because the next 16 bits are the same as the first 16 bits of the *l*-mer 8 positions to the right of the current one. Therefore, the table of compressed *l*-mers only requires *O*(*n*(*m*-*l*+1)) words of memory.

#### Preprocess distances for pairs of *l*-mers

The filtering step tests many times if two *l*-mers have a distance of no more than 2*d*. Thus, for every pair of *l*-mers we compute this bit of information in advance.

#### Cache locality

We can update *R* in an efficient manner as follows. Every row in the updated matrix *R*^′^ is a subset of the corresponding row in the current matrix *R*, because some elements will be filtered out. Therefore, we can store *R*^′^ in the same memory locations as *R*. To do this, in each row, we move the elements belonging to *R*^′^ at the beginning of the row. In addition, we keep track of how many elements belong to *R*^′^. To revert from *R*^′^ back to *R*, we restore the row sizes to their previous values. The row elements will be the same even if they have been moved within the row. The same process can be repeated at every step of the recursion, therefore the whole “stack” of *R* matrices is stored in a single matrix. This reduces the memory requirement and improves cache locality. The cache locality is improved because at every step of the recursion, in each row, we access a subset of the elements we accessed in the previous step, and those elements are in contiguous locations of memory.

#### Find motifs for a subset of the strings

We use the speedup technique described in [10]: compute the motifs for *n*^′^<*n* of the input strings, then test each of them against the remaining *n*-*n*^′^ strings. For the results in this paper *n*^′^ was heuristically computed using the formula provided in Appendix 1.

### Memory and Runtime

Since we store all matrices *R* in the space of a single matrix they only require *O*(*n*(*m*-*l*+1)) words of memory. To this we add *O*(*n*^2^) words to store row sizes for the at most *n* matrices which share the same space. The bits of information for compatible *l*-mer pairs take *O*((*n*(*m*-*l*+1))^2^/*w*) words, where *w* is the number of bits in a machine word. The table of compressed *l*-mers takes *O*(*n*(*m*-*l*+1)) words. Therefore, the total memory used by the algorithm is *O*(*n*(*n*+*m*-*l*+1)+(*n*(*m*-*l*+1))^2^/*w*).

The more time we spend in the sample driven part, the less time we have to spend in the pattern driven part and vice-versa. Ideally we want to choose the threshold where we switch between the two parts such that their runtimes are almost equal. The optimal threshold can be determined empirically by running the algorithm on a small subset of the tuples. In practice, PMS8 heuristically estimates the threshold *t* such that it increases with *d* and |*Σ*| to avoid generating very large neighborhoods and it decreases with *m* to avoid spending too much time on filtering. All the results reported in this paper have been obtained using the default threshold estimation provided in Appendix 1.

### Parallel implementation

We can think of *m*-*l*+1 independent sub problems, one for each *l*-mer in the first string. The first string in each sub problem is an *l*-mer of the original first string and the rest of the strings are the same as in the original input. Because of this, the problem seems to be “embarrassingly parallel.” A straightforward parallelization idea is to assign an equal number of subproblems to each processor. This method has the advantage that no inter-processor communication is necessary beyond broadcasting the input to all processors. This method would work well for algorithms where each subproblem is expected to have a similar runtime. However, for PMS8, the runtime of each subproblem is very sensitive to the size of the neighborhoods for various combinations of *l*-mers and therefore some processors may end up starving while others are still busy.

To alleviate the above shortcoming we employ the following strategy. The processor with rank 0 is a scheduler and the others are workers. The scheduler spawns a separate worker thread to avoid using one processor just for scheduling. The scheduler reads the input and broadcasts it to all workers. Then each worker requests a sub problem from the scheduler, solves it and repeats. The scheduler loops until all jobs have been requested and all workers have been notified that no more jobs are available. At the end, all processors send their motifs to the scheduler. The scheduler loops through all the processors and collects the results. The scheduler then outputs the results.

### Pruning conditions

In this section we present pruning conditions applied for filtering *l*-mers in the sample driven part and for pruning enumeration trees in the pattern driven part.

Two *l*-mers *a* and *b* have a common neighbor *M* such that *Hd*(*a*,*M*)≤*d*_*a*_ and *Hd*(*b*,*M*)≤*d*_*b*_ if and only if *Hd*(*a*,*b*)≤*d*_*a*_+*d*_*b*_. For 3 *l*-mers, no trivial necessary and sufficient conditions have been known up to now. In [8] sufficient conditions for 3 *l*-mers are obtained from a preprocessed table. However, as *l* increases the memory requirement of the table becomes a bottleneck. We will give simple necessary and sufficient conditions for 3 *l*-mers to have a common neighbor. These conditions are also necessary for more than 3 *l*-mers.

Let *T* be a set of *l*-mers and *M* be an *l*-mer. If ∑*u*∈*THd*(*M*,*u*)>|*T*|*d* then, by the pigeonhole principle, one *l*-mer must have a distance from *M* greater than *d*. Therefore, *M* cannot be a common neighbor of the *l*-mers in *T*. If we have a lower bound on ∑*u*∈*THd*(*M*,*u*) for any *M*, then we can use it as a pruning condition. If the lower bound is greater than |*T*|*d* then there is no common neighbor for *T*. One such lower bound is the *consensus total distance*.

#### Definition 1

Let *T* be a set of *l*-mers, where *k*=|*T*|. For every *i*, the set *T*_1_[*i*],*T*_2_[*i*],..,*T*_*k*_[*i*] is called the *i*-th column of *T*. Let *m*_*i*_ be the maximum frequency of any character in column *i*. Then *Cd*(*T*)= ∑*i*=1..*lk*-*m*_*i*_ is called the consensus total distance of *T*.

The consensus total distance is a lower bound for the total distance between any *l*-mer *M* and the *l*-mers in *T* because, regardless of *M*, the distance contributed by column *i* to the total distance is at least *k*-*m*_*i*_. The consensus total distance for a set of two *l*-mers *A* and *B* will be denoted by *Cd*(*A*,*B*). Also notice that *Cd*(*A*,*B*)=*Hd*(*A*,*B*). We can easily prove the following lemma.

#### Lemma 1

Let *T* be a set of *l*-mers and *k*=|*T*|. Let *d*_1_,*d*_2_,…*d*_*k*_ be non-negative integers. There exists a *l*-mer *M* such that *Hd*(*M*,*T*_*i*_)≤*d*_*i*_,∀*i*, only if $\text{Cd}(T)\leq \Sigma_{i=1}^{k}d_{i}$.

#### Theorem 1

Let *T* be a set of 3 *l*-mers and *d*_1_,*d*_2_,*d*_3_ be non-negative integers. There exists a *l*-mer *M* such that *Hd*(*M*,*T*_*i*_)≤*d*_*i*_,∀*i*,1≤*i*≤3 if and only if the following conditions hold:

**i)***Cd*(*T*_*i*_,*T*_*j*_)≤*d*_*i*_+*d*_*j*_,∀*i*,*j*,1≤*i*<*j*≤3

**ii)***Cd*(*T*)≤*d*_1_+*d*_2_+*d*_3_

#### *Proof*

The “only if” part follows from lemma 1.

**Figure 1:**
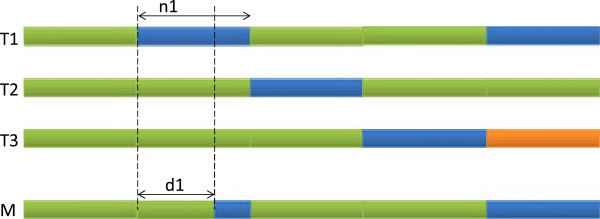
**Proof of theorem 1, case 1.** Proof of theorem 1, case 1: There exists *i*,1≤*i*≤3 for which *n*_*i*_≥*d*_*i*_. Without loss of generality we assume *i*=1. The top 3 rows represent the input *l*-mers. The last row shows a common neighbor *M*. In any column, identical colors represents matches, different colors represent mismatches.

For the “if” part we show how to construct a common neighbor *M* provided that the conditions hold. We say that a column *k* where *T*_1_[ *k*]=*T*_2_[ *k*]=*T*_3_[ *k*] is of type *N*_0_. If *T*_1_[ *k*]≠*T*_2_[*k*]=*T*_3_[ *k*] then the column is of type *N*_1_. If *T*_1_[ *k*]=*T*_3_[ *k*]≠*T*_2_[ *k*] the column is of type *N*_2_ and if *T*_1_[ *k*]=*T*_2_[ *k*]≠*T*_3_[ *k*] then the column is of type *N*_3_. If all three characters in the column are distinct, the column is of type *N*_4_. Let *n*_*i*_,∀*i*,0≤*i*≤4 be the number of columns of type *N*_*i*_. Consider two cases:

Case 1) There exists *i*,1≤*i*≤3 for which *n*_*i*_≥*d*_*i*_. We construct *M* as illustrated in Figure 1. Pick *d*_*i*_ columns of type *n*_*i*_. For each chosen column *k* set *M*[ *k*]=*T*_*j*_[ *k*] where *j*≠*i*. For all other columns set *M*[ *k*]=*T*_*i*_[ *k*]. Therefore *Cd*(*T*_*i*_,*M*)=*d*_*i*_. For *j*≠*i* we know that *Cd*(*T*_*i*_,*T*_*j*_)≤*d*_*i*_+*d*_*j*_ from condition **i)** (condition **i** is assumed to be true at this point because we are proving the “if” part). We also know that *Cd*(*T*_*i*_,*M*)+*Cd*(*M*,*T*_*j*_)≤*Cd*(*T*_*i*_,*T*_*j*_) from the triangle inequality. It follows that *Cd*(*M*,*T*_*j*_)≤*d*_*j*_. Since *Cd*(*M*,*T*_*j*_)=*Hd*(*M*,*T*_*j*_) it means that *M* is indeed a common neighbor of the three *l*-mers.

**Figure 2:**
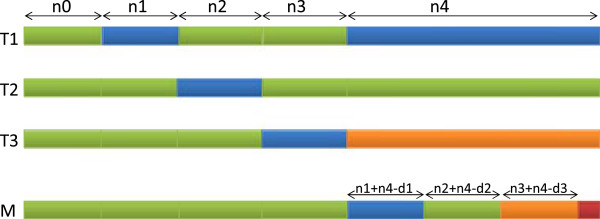
**Proof of theorem 1, case 2.** Proof of theorem 1, case 2: *n*_*i*_<*d*_*i*_ for all *i*, 1≤*i*≤3. The top 3 rows represent the input *l*-mers. The last row shows a common neighbor *M*. In any column, identical colors represents matches, different colors represent mismatches.

Case 2) For all *i*,1≤*i*≤3 we have *n*_*i*_<*d*_*i*_. We construct *M* as shown in Figure 2. For columns *k* of type *N*_0_,*N*_2_ and *N*_3_ we set *M*[ *k*]=*T*_1_[ *k*]. For columns of type *N*_1_ we set *M*[ *k*]=*T*_2_[ *k*]. For any *i*,1≤*i*≤3 the following applies. If *n*_*i*_+*n*_4_≤*d*_*i*_ then the Hamming distance between *M* and *T*_*i*_ is less than *d*_*i*_ regardless of what characters we choose for *M* in the columns of type *N*_4_. On the other hand, if *n*_*i*_+*n*_4_>*d*_*i*_ then *M* and *T*_*i*_ have to match in at least *n*_*i*_+*n*_4_-*d*_*i*_ columns of type *N*_4_. Thus, we pick *max*(0,*n*_*i*_+*n*_4_-*d*_*i*_) columns of type *N*_4_ and for each such column *k* we set *M*[ *k*]=*T*_*i*_[ *k*]. Now we prove that we actually have enough columns to make the above choices, in other words $\Sigma_{i=1}^{3}\text{max}(0,n_{i}+n_{4}-d_{i})\leq n_{4}$. This is equivalent to the following conditions being true: 

**a)**For any *i*,1≤*i*≤3 we want *n*_*i*_+*n*_4_-*d*_*i*_≤*n*_4_. This is true because *n*_*i*_<*d*_*i*_.

**b)**For any *i*,*j*,1≤*i*<*j*≤3 we want (*n*_*i*_+*n*_4_-*d*_*i*_)+(*n*_*j*_+*n*_4_-*d*_*j*_)≤*n*_4_. This can be rewritten as *n*_*i*_+*n*_*j*_+*n*_4_≤*d*_*i*_+*d*_*j*_. The left hand side is *Hd*(*T*_*i*_,*T*_*j*_) which we know is less or equal to *d*_*i*_+*d*_*j*_.

**c)**We want $\Sigma_{i=1}^{3}n_{i}+n_{4}-d_{i}\leq n_{4}$. This can be rewritten as *n*_1_+*n*_2_+*n*_3_+2*n*_4_≤*d*_1_+*d*_2_+*d*_3_. The left hand side is *Cd*(*T*) which we know is less than *d*_1_+*d*_2_+*d*_3_.

One of our reviewers kindly pointed out that the above proof is similar to an algorithm in [11].

## Results and discussion

PMS8 is implemented in C++ and uses OpenMPI for communication between processors. PMS8 was evaluated on the Hornet cluster in the Booth Engineering Center for Advanced Technology (BECAT) at University of Connecticut. The Hornet cluster consists of 64 nodes, each equipped with 12 Intel Xeon X5650 Westmere cores and 48 GB of RAM. The nodes use Infiniband networking for MPI. In our experiments we employed at most 48 cores on at most 4 nodes.

**Figure 3:**
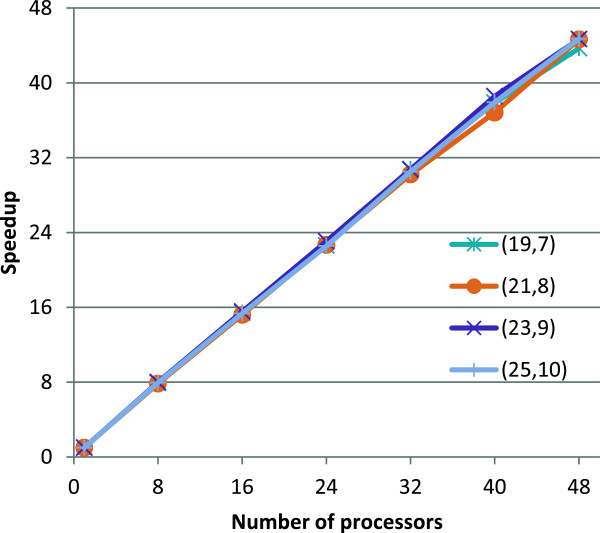
**Speedup of the parallel implementation over the single core version.** Speedup of the multi-core version of PMS8 over the single core version, for several datasets.

We generated random (*l*,*d*) instances according to [1] and as described in the introduction. For every (*l*,*d*) combination we report the average runtime over 5 random instances. For several challenging instances, in Figure 3 we present the speedup obtained by the parallel version over the single core version. For *p*=48 cores the speedup is close to *S*=45 and thus the efficiency is *E*=*S*/*p*=94*%*.

**Figure 4:**
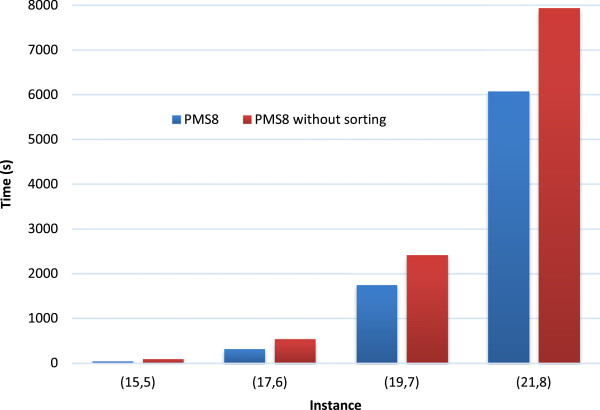
**The effect of the “sort rows by size” speedup on runtime.** Comparison between PMS8 and PMS8 without sorting rows by size (see the section on Speedup techniques). Note that, in both versions, all the other speedups are enabled. The runtimes are for single core execution, averaged over 5 random instances.

In Figure 4 we show the effect of the first speedup technique (sorthing rows by size) on the runtime. Note that all other speedups are enabled, only sorting rows by size is varied. The figure shows that sorting the rows by size improves the speed of PMS8 by 25% to 50%.

**Figure 5:**
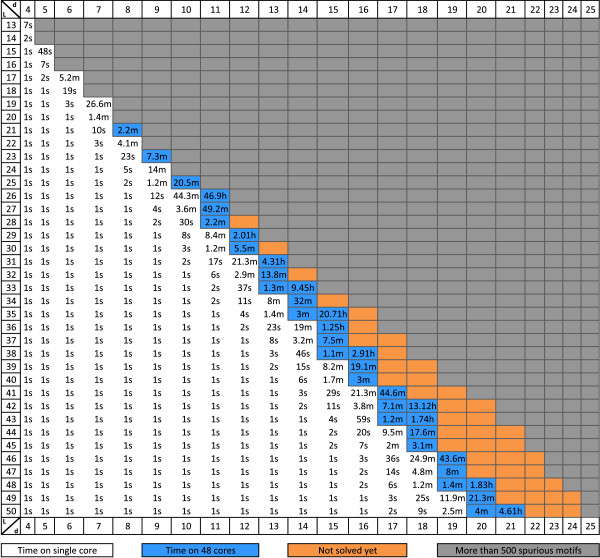
**PMS8 runtimes for multiple combinations of *l* and *d*.** PMS8 runtimes for datasets with *l* up to 50 and *d* up to 25 averaged over 5 random datasets. White background signifies single core execution. Blue background signifies execution using 48 cores. Instances in gray have more than 500 spurious motifs. Orange cells indicate unsolved instances. Time is reported in hours (h), minutes (m) and seconds (s).

The runtime of PMS8 on instances with *l* up to 50 and *d* up to 21 is shown in Figure 5. Instances which are expected to have more than 500 motifs simply by random chance (spurious motifs) are excluded. The expected number of spurious motifs was computed as described in Appendix 1. Instances where *d* is small relative to *l* are solved efficiently using a single CPU core. For more challenging instances we report the time taken using 48 cores.

**Figure 6:**
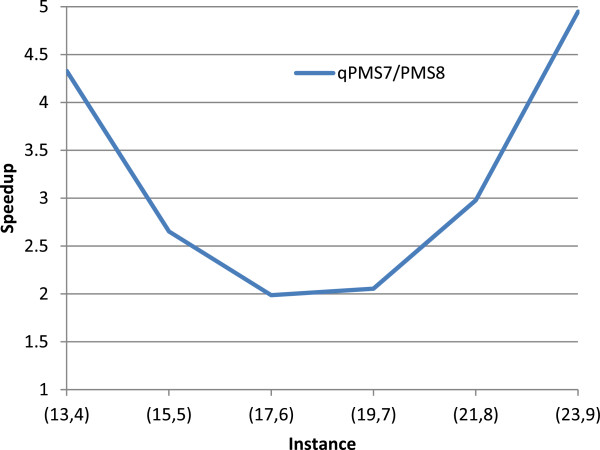
**Speedup of PMS8 single core over qPMS7.** Ratio of runtimes between qPMS7 and PMS8 running on a single core. Both programs have been executed on the same hardware and the same datasets. The times are average runtimes over 5 instances for each dataset.

A comparison between PMS8 and qPMS7 [5] on challenging instances is shown in Table 1. Both programs have been executed on the Hornet cluster. qPMS7 is a sequential algorithm. PMS8 was evaluated using up to 48 cores. The speedup of PMS8 single core over qPMS7 is shown in Figure 6. The speedup is high for small instances because qPMS7 has to load an ILP table. For larger instances the speedup of PMS8 sharply increases. This is expected because qPMS7 always generates neighborhoods for tuples of 3 *l*-mers, which become very large as *l* and *d* grow. On the other hand, PMS8 increases the number of *l*-mers in the tuple with the instance size. With each *l*-mer added to the tuple, the size of the neighborhood reduces exponentially, whereas the number of neighborhoods generated increases by a linear factor. The ILP table precomputation requires solving many ILP formulations. The table then makes qPMS7 less memory efficient than PMS8. The peak memory used by qPMS7 for the challenging instances in Table 1 was 607 MB whereas for PMS8 it was 122 MB. Furthermore, due to the size of the ILP table, qPMS7 is not able to solve any instances where *l*>23. PMS8 is the first algorithm to solve the challenging instances (25,10) and (26,11).

**Table 1:** Comparison between qPMS7 and PMS8

**Instance**	**qPMS7**	**PMS8**^**1**^	**PMS8**^**16**^	**PMS8**^**32**^	**PMS8**^**48**^
(13,4)	29s	7s	3s	2s	2s
(15,5)	2.1m	48s	5s	4s	3s
(17,6)	10.3m	5.2m	22s	12s	9s
(19,7)	54.6m	26.6m	1.7m	52s	37s
(21,8)	4.87h	1.64h	6.5m	3.3m	2.2m
(23,9)	27.09h	5.48h	21.1m	10.7m	7.4m
(25,10)	-	15.45h	1.01h	30.4m	20.7m
(26,11)	-	-	-	-	46.9h

Comparison between qPMS7 and PMS8 on challenging instances. PMS8^*P*^ means PMS8 used *P* CPU cores. Both programs have been executed on the same hardware and the same datasets. The times are average runtimes over 5 instances for each dataset.

Some recent results in the literature have also focused on instances other than the challenging ones presented above. A summary of these results and a comparison with PMS8 is presented in Table 2, starting with the most recent results. These results have been obtained on various types of hardware: single core, multi-core, GPU, grid. In the comparison, we try to match the number of processors whenever possible. The speed difference is of several orders of magnitude in some cases which indicates that the pruning conditions employed by PMS8 exponentially reduce the search space compared to other algorithms.

**Table 2:** Comparison between PMS8 and recent results in the literature

**Previous algorithm**	**Instance**	**Time**	**Cores**	**PMS8 time**	**PMS8 cores**
Abbas et al. 2012 [12], PHEP_PMSprune	(21,8)	20.42h	8	6.5m	1
Yu et al. 2012 [3], PairMotif	(27, 9)	10h	1	4s	1
Desaraju and Mukkamala 2011 [7]	(24,6)	347s	1	1s	1
	(48,12)	188s	1	1s	1
Dasari et al. 2011 [13], mSPELLER / gSPELLER	(21,8)	3.7h	16	6.5m	16
	(21,8)	2.2h	4 GPUs x 240 cores	6.5m	16
Dasari et al. 2010 [14], BitBased	(21,8)	1.1h		6.5m	16
Dasari and Desh 2010 [15], BitBased	(21,8)	6.9h	16	6.5m	16
Sahoo et al. 2011 [16]	(16,4)	106s	4	1s	1
Sun et al. 2011 [17], TreeMotif	(40,14)	6h	1	6s	1
He et al. 2010 [18], ListMotif	(40,14)	28,087s	1	6s	1
Faheem 2010 [19], skip-Brute Force	(15,4)	2934s	96 nodes	1s	1
Ho et al. 2009 [6], iTriplet	(24,8)	4h	1	5s	1
	(38,12)	1h	1	1s	1
	(40,12)	5m	1	1s	1

Side by side comparison between PMS8 and recent results in the literature. Time is reported in seconds (s), minutes (m) or hours (h). Note that the hardware is different, though we tried to match the number of processors when possible. Also, the instances are randomly generated using the same algorithm, however the actual instances used by the various papers are most likely different. For PMS8, the times are averages over 5 randomly generated instances.

We also compared PMS8 with qPMS7 on the real datasets discussed in [20]. We excluded datasets with less than 4 input sequences because these are not very challenging. For each dataset we chose two combinations of *l* and *d*. These combinations were chosen on a dataset basis because for large values of *d* the number of reported motifs is excessive and for small values of *d* the instance is not very challenging. To make qPMS7 behave like PMS8 we set the quorum percent to 100% (*q*=*n*). In Table 3 we report the dataset name, the total number of sequences, the total number of bases in each dataset, the *l* and *d* combination and the runtimes of the two algorithms. Note that both algorithms are exact algorithms and therefore the sensitivity and specificity are the same. Similar to the comparison on synthetic data, the comparison on real data reveals that PMS8 outperforms qPMS7.

**Table 3:** **Runtime comparison between PMS8 and qPMS7 on real datasets from [**[20]**]**

**Dataset**	**n**	**Total no. bases**	**l**	**d**	**PMS8 time**	**qPMS7 time**
dm01r	4	6000	21	4	1	55
dm01r	4	6000	23	5	1	6
dm04r	4	8000	21	4	1	5
dm04r	4	8000	23	5	1	5
hm01r	18	36000	21	6	10	14
hm01r	18	36000	23	7	25	40
hm02r	9	9000	21	6	1	11
hm02r	9	9000	23	7	4	35
hm03r	10	15000	21	6	3	24
hm03r	10	15000	23	7	14	146
hm04r	13	26000	21	6	6	44
hm04r	13	26000	23	7	15	39
hm05r	3	3000	21	4	1	6
hm05r	3	3000	23	5	1	46
hm08r	15	7500	17	5	1	7
hm08r	15	7500	17	6	46	251
hm19r	5	2500	23	5	1	5
hm19r	5	2500	23	6	1	5
hm20r	35	70000	21	6	27	32
hm20r	35	70000	23	7	56	136
hm26r	9	9000	23	6	1	5
hm26r	9	9000	23	7	5	46
mus02r	9	9000	21	6	1	11
mus02r	9	9000	23	7	2	45
mus04r	7	7000	21	6	1	15
mus04r	7	7000	23	7	2	22
mus05r	4	2000	21	5	1	79
mus05r	4	2000	23	6	1	5
mus07r	4	6000	21	5	1	79
mus07r	4	6000	23	5	1	6
mus10r	13	13000	21	6	2	56
mus10r	13	13000	23	7	2	70
mus11r	12	6000	21	7	8	150
mus11r	12	6000	23	8	23	938
yst01r	9	9000	21	6	2	14
yst01r	9	9000	23	7	8	63
yst02r	4	2000	21	5	1	5
yst02r	4	2000	23	6	1	6
yst03r	8	4000	21	6	1	8
yst03r	8	4000	23	7	1	19
yst04r	6	6000	21	4	1	5
yst04r	6	6000	23	5	1	5
yst05r	3	1500	21	4	1	5
yst05r	3	1500	23	5	1	5
yst06r	7	3500	21	6	1	6
yst06r	7	3500	23	7	2	12
yst08r	11	11000	21	5	1	6
yst08r	11	11000	23	6	1	6
yst09r	16	16000	21	6	2	17
yst09r	16	16000	23	7	6	68

For each dataset we tested two combinations of *l* and *d*. For qPMS7 we set *q* =*n*. Both algorithms were executed on a single CPU core. Time is reported in seconds, rounded up to the next second.

## Conclusions

We have presented PMS8, an efficient algorithm for the PMS problem. PMS8 is able to efficiently generate neighborhoods for *tl*-mers at a time, by using the pruning conditions presented in this paper. Previous algorithms generate neighborhoods for only up to three *l*-mers at a time whereas in PMS8 the value of *t* is increased as the instances become more challenging and therefore the exponential explosion is postponed. The second reason for the efficiency of PMS8 comes from the careful implementation which employs several speedup techniques and emphasizes cache locality.

## Appendix

### Appendix 1 Generating neighborhoods

**Algorithm 1. GenerateNeighborhood**(*T*,*d*)

### Appendix 2 PMS8 pseudocode

**Algorithm 2. PMS8**(*T*,*d*)

### Appendix 3 Challenging instances

For a fixed *l*, as *d* increases, the instance becomes more challenging. However, as *d* increases, the number of false positives also increases, because many motifs will appear simply by random chance. The expected number of spurious motifs in a random instance can be estimated as follows (see e.g., [4]). The number of *l*-mers in the neighborhood of a given *l*-mer *M* is $N(\Sigma ,l,d)=\Sigma_{i=0}^{d}{(}_{d}^{l}){\left(|\Sigma |-1\right)}^{d}$. The probability that *M* is a *d*-neighbor of a random *l*-mer is *p*(*Σ*,*l*,*d*)=*N*(*Σ*,*l*,*d*)/|*Σ*|^*l*^. The probability that *M* has at least one *d*-neighbor among the *l*-mers of a string of length *m* is thus *q*(*m*,*Σ*,*l*,*d*)=1-(1-*p*(*Σ*,*l*,*d*))^*m*-*l*+1^. The probability that *M* has at least one *d*-neighbor in each of *n* random strings of length *m* is *q*(*m*,*Σ*,*l*,*d*)^*n*^. Finally, the expected number of spurious motifs in an instance with *n* strings of length *m* each is: |*Σ*|^*l*^*q*(*m*,*Σ*,*l*,*d*)^*n*^. In this paper we consider all combinations of *l* and *d* where *l* is at most 50 and the number of spurious motifs (expected by random chance) does not exceed 500. Note that for a fixed *d*, if we can solve instance (*l*,*d*) we can also solve all instances (*l*^′^,*d*) where *l*^′^>*l*, because they are less challenging than (*l*,*d*).

### Appendix 4 Heuristics for t and n

In the methods section we mentioned that we heuristically estimate the threshold *t* at which we switch from the pattern driven to the sample driven part. The exact formula used by the algorithm to compute *t* was $t=\max(2,\lfloor \sqrt{2(d+1)\log\Sigma -\log m}\rfloor )$. This follows the intuition that *t* should increase with *Σ* to avoid large neighborhoods and decrease with *m* to avoid spending too much time on filtering.

In the Speedup techniques section we mentioned a speedup where we compute motifs for a subset of *n*^′^<*n* strings. By default, the algorithm heuristically computes *n*^′^ as *n*^′^=*min*(*n*,*t*+*n*/4-*logt*). These simple heuristics worked well enough on all our test cases, however the user can easily override them.

## Competing interests

The authors declare that they have no competing interests.

## Authors’ contributions

MN and SR designed and analyzed the algorithms. MN implemented the algorithms and carried out the empirical experiments. MN and SR analyzed the empirical results and drafted the manuscript. All authors read and approved the final manuscript.
